# Ovarian Drilling in PCOS: Is it Really Useful?

**DOI:** 10.3389/fsurg.2015.00030

**Published:** 2015-07-17

**Authors:** Issam Lebbi, Riadh Ben Temime, Anis Fadhlaoui, Anis Feki

**Affiliations:** ^1^Ob-Gyn and Fertility Private Clinic, Dream Center, Tunis, Tunisia; ^2^Department of Obstetrics and Gynecology, Charles Nicolle Hospital, Tunis, Tunisia; ^3^Department of Obstetrics and Gynecology, HFR Fribourg – Hôpital Cantonal, Fribourg, Switzerland

**Keywords:** anovulation, polycystic ovary syndrome, ovarian infertility, ovarian drilling, laparoscopic drilling

## Abstract

Polycystic ovary syndrome (PCOS) is a frequent disorder, affecting approximately 5–10% of infertile women. It can represent more than 80% of cases of infertility due to anovulation. The main goal of treatment is the induction of mono-ovulatory cycles. A pragmatic management of infertility in PCOS will allow most patients to conceive. Weight loss and clomiphene citrate (CC) are the first-line components of patients treatment before gonadotrophins are used. However, during gonadotrophin administration, there is a high risk of ovarian hyper-stimulation and multiple pregnancies. So, surgery with laparoscopic ovarian drilling is often used before gonadotrophins in order to obtain normal ovulatory cycles.

Polycystic ovary syndrome (PCOS) is a frequent disorder, affecting 5–10% of infertile women (1, 2). It is responsible for more than 80% of cases of infertility due to anovulation (3).

The main goal of treatment is the induction of mono-ovulatory cycles.

A “stepwise approach” to the management of infertility in PCOS will permit most patients to achieve pregnancy and a live birth (4):

1. Appropriate living style, such as diet and physical exercises in order to reduce weight.
2. Oral medication agents:
  ○ The first-line oral agents include clomiphene citrate (CC) (selective estrogen receptor modulators) with 49% of ovulation rate, 30% of pregnancy rate, and 23% of live birth rate at 6 months. Therefore, there is an increased rate of multiple gestation: 8%.
  ○ The other first-line oral agent is Letrozole (aromatase inhibitors) with recent evidence recommending the use of letrozole [in Ref. (4)]:
    – the ovulation rate is 61.7% for letrozole versus 48.3% for CC, *p* < 0.0001;
    – the live birth rate is 27.5% for letrozole versus 19.2% for CC, *p* = 0.007;
    – there is 44% higher live birth rate with letrozole in patients with high body mass index (BMI) and longstanding infertility.
  ○ Metformin (insulin sensitizers) is an adjunct to induction of ovulation in patients with glucose intolerance and obesity.
    For women with WHO Group II ovulation disorders who are known to be resistant to CC, both the agency for Healthcare Research and Quality (AHRQ) and the National Institute for Health and Clinical Excellence (NICE) consider one of the following second-line treatments, depending on clinical circumstances and the woman’s preference:
    - laparoscopic ovarian drilling (LOD),
    - combined treatment with CC and metformin if not already offered as first-line treatment, or
    - Gonadotrophins (5, 6).
3. Gonadotrophins are the second-line treatment in case of CC resistance or CC failure (no pregnancy after four to six ovulatory cycles) (5, 7)
4. The LOD may be considered as a second-line treatment in a selected population (3, 4). LOD may be considered in women with CC-resistant PCOS, particularly when there are other indications for laparoscopy, if there is a high risk of multiple pregnancies or a contra-indication of multiple pregnancies. (5, 7).
5. *In vitro* fertilization (IVF) is indicated in the treatment of PCOS-associated infertility with high success rates and potentially lower rate of multiple gestations if it is well managed. Indeed, careful monitoring of controlled ovulation aims to avoid multiple pregnancies when using gonadotrophins in IVF (4).

In summary, weight loss and CC are the first-line components of patients’ treatment before gonadotrophins use (5–7). However, during gonadotrophin administration, there is a high risk of ovarian hyper-stimulation (OHSS) and multiple pregnancies. The risk of multiple pregnancies after LOD is lower than for gonadotrophin stimulation (4). So, surgery with LOD may be an alternative before gonadotrophins in order to obtain normal ovulatory cycles (5, 7).

Almost three decades after the first report of LOD using a unipolar electrode by Halvard Gjönnaess (2), it was proposed as a less invasive alternative than bilateral ovarian wedge resection; till now, many controversies are still not clarified concerning ovarian drilling mechanism of action and what is the best and cost-effective technique in the treatment of PCOS syndrome.

The most plausible mechanisms of action are the destruction of ovarian follicles and a part of the ovarian stroma, inducing a reduction of serum androgens and inhibin levels, which results in an increase of FSH and restores the ovulation function (1). LOD may also increase ovarian blood flow, allowing a high delivery of gonadotrophins and post-surgical local growth factors. An improvement of insulin sensitivity after LOD has also been suggested (1, 4, 8, 9).

The common technique of LOD is the use of monopolar electrocautery (diathermy) or laser with comparable results (8–12).

Normally, three to eight diathermy punctures are performed in each ovary using 600–800 J energy for each puncture, lead to further normal ovulation in 74% of the cases in the next 3–6 months. More than eight punctures seem to increase the occurrence of post-operative pelvic adhesions and decrease the ovarian reserve (8).

Different other minimally invasive techniques were later described for ovarian drilling. Some authors proposed LOD using a bipolar energy probe as a potentially safer method compared to unipolar energy. Other authors described the micro-laparoscopic ovarian drilling technique (MLOD) under local anesthesia, which allows outpatient management without general anesthesia (13).

Fertiloscopy (transvaginal hydrolaparoscopy) was also described as a technique with comparable results to those of laparoscopy (14).

Laser was also tested in laparoscopy or fertiloscopy for ovarian drilling with the same results as monopolar needle.

A systematic Cochrane review including 25 randomized controlled trials of sub-fertile women with clomiphene-resistant PCOS who undertook LOD in order to induce ovulation concluded that there was no evidence of a significant difference in rates of clinical pregnancy, live birth, or miscarriage in clomiphene-resistant PCOS women undergoing LOD compared to other medical treatments.

The reduction of multiple pregnancy rates in women undergoing LOD makes this technique attractive and useful (8).

In a comprehensive review of ovarian drilling for PCOS, Fernandez (15) concluded that ovarian drilling leads to spontaneous restoration of fertility in 20–64% of women with PCOS who had previously been infertile as a result of anovulation and who did not respond to CC treatment, while the meta-analysis by Campo (16) reported a narrower range of success in 44–50% of patients. Several factors could influence the efficacy of ovarian drilling: a higher likelihood of success in patients with elevated LH concentrations (>10 IU/l) and <3 years of infertility. However, the influence of other factors, such as BMI, insulin resistance, and testosterone concentrations, is contradictory (15, 17).

The results of LOD are not superior to CC as a first-line treatment of ovulation induction in women with PCOS. Furthermore, there is no significant difference in pregnancy and live birth rates per women undergoing LOD versus six cycles of CC as a first-line approach for anovulatory infertile patients. But, in women with failure to conceive after six to nine cycles of CC, LOD is the best choice to induce mono-ovulatory cycles with higher pregnancy rate. The mechanism is that LOD avoids peripheral anti-estrogenic effects of CC on both endometrium and cervical mucus and the hypersecretion of LH leading to premature luteinization in response to CC responsible for its failure (1, 18, 19).

Several studies have reported that LOD prior to ART is beneficial in decreasing the risk of severe OHSS and increasing the “take home baby “ rate in women who have previously had canceled IVF cycles due to OHSS risk or who suffered from OHSS in a previous treatment. This finding may be attributed to a reduced ovarian blood flow velocity and serum vascular endothelial growth factor (VEGF) concentration after LOD (9).

In conclusion, all meta-analysis confirmed that LOD is a second-line treatment in PCOS patients, especially those with CC resistance (8, 9). The main benefits are shorter time to pregnancy and less need to ovulation induction drugs. The other advantages of this technique are more comfort, cost-effectiveness, and possibility to be performed ambulatory.

However, the results of LOD are not better than those of CC as a first-line treatment in PCOS.

## Conflict of Interest Statement

The authors declare that the research was conducted in the absence of any commercial or financial relationships that could be construed as a potential conflict of interest.
